# Discrimination of Nonalcoholic Steatohepatitis Using Transient Elastography in Patients with Nonalcoholic Fatty Liver Disease

**DOI:** 10.1371/journal.pone.0157358

**Published:** 2016-06-10

**Authors:** Hye Won Lee, Soo Young Park, Seung Up Kim, Jae Young Jang, Hana Park, Ja Kyung Kim, Chun Kyon Lee, Young Eun Chon, Kwang-Hyub Han

**Affiliations:** 1 Department of Internal Medicine, Institute of Gastroenterology, Yonsei University College of Medicine, Seoul, Korea; 2 Department of Internal Medicine, Kyungpook National University Hospital, Kyungpook National University College of Medicine, Seoul, Korea; 3 Department of Internal Medicine, College of Medicine, Soonchunhyang University, Seoul, Korea; 4 Department of Internal Medicine, CHA Bundang Medical Center, CHA University, Bundang, Korea; 5 National Health Insurance Cooperation, Ilsan Hospital, Ilsan, Korea; Taipei Veterans General Hospital, TAIWAN

## Abstract

**Background/aims:**

The accuracy of noninvasive markers to discriminate nonalcoholic steatohepatitis (NASH) is unsatisfactory. We investigated whether transient elastography (TE) could discriminate patients with NASH from those with nonalcoholic fatty liver disease (NAFLD).

**Methods:**

The patients suspected of NAFLD who underwent liver biopsy and concomitant TE were recruited from five tertiary centers between November 2011 and December 2013.

**Results:**

The study population (n = 183) exhibited a mean age of 40.6 years and male predominance (n = 111, 60.7%). Of the study participants, 89 (48.6%) had non-NASH and 94 (51.4%) had NASH. The controlled attenuation parameter (CAP) and liver stiffness (LS) were significantly correlated with the degrees of steatosis (r = 0.656, *P*<0.001) and fibrosis (r = 0.714, *P*<0.001), respectively. The optimal cut-off values for steatosis were 247 dB/m for S1, 280 dB/m for S2, and 300 dB/m for S3. Based on the independent predictors derived from multivariate analysis [*P* = 0.044, odds ratio (OR) 4.133, 95% confidence interval (CI) 1.037–16.470 for *C*AP>250 dB/m; *P* = 0.013, OR 3.399, 95% CI 1.295–8.291 for *L*S>7.0 kPa; and *P*<0.001, OR 7.557, 95% CI 2.997–19.059 for *A*lanine aminotransferase>60 IU/L], we developed a novel CLA model for discriminating patients with NASH. The CLA model showed good discriminatory capability, with an area under the receiver operating characteristic curve (AUROC) of 0.812 (95% CI 0.724–0.880). To assess discriminatory power, the AUROCs, as determined by the bootstrap method, remained largely unchanged between iterations, with an average value of 0.833 (95% CI 0.740–0.893).

**Conclusion:**

This novel TE-based CLA model showed acceptable accuracy in discriminating NASH from simple steatosis. However, further studies are required for external validation.

## Introduction

The prevalence of nonalcoholic fatty liver disease (NAFLD) is growing worldwide. The prevalence of NAFLD ranges from 6.3% to 33%, with a median of 20% in the general population [1]. According to recent data, 68% of adults are overweight or obese, and NAFLD affects approximately 30% of the US population [2]. The prevalence of NAFLD in Asia has also increased and is associated with a Westernized diet, lifestyle changes, and a lack of exercise. NAFLD in the Asian population was estimated at 15–45% [3]. In South Korea, the prevalence of NAFLD diagnosed by ultrasonography was high, ranging from 16.1% to 33.3%. Even among healthy living donors, the prevalence of NAFLD approaches 20–51% [4,5].

The histologic spectrum of NAFLD includes nonalcoholic fatty liver (NAFL) as simple steatosis and nonalcoholic steatohepatitis (NASH). NASH is defined as hepatic steatosis and inflammation with hepatocyte injury with or without fibrosis, potentially progressing to fibrosis and ultimately cirrhosis [6]. The prevalence of NASH is estimated at 3–5% [1]. Among obese subjects, the prevalence of NAFL is reportedly 60%, with NASH approaching 20–25% [7]. Several reports have shown that the progression to fibrosis and cirrhosis in NASH are 25% and 15% over 5 years [8,9]. Both 5- and 10-year survival rates of patients with NASH are reported to be 67% and 59%, respectively [7]. Therefore, an accurate diagnosis of NASH, which shows a poor prognosis, is important in predicting the long-term prognosis of patients with NAFLD.

Liver biopsy (LB) is still the standard test to diagnosis NAFLD and the presence of early liver fibrosis. However, histologic lesions are not evenly distributed throughout the liver [10,11]. A sampling error is the biggest limitation in the diagnosis of NAFLD by LB [11], with inflammatory lesions and ballooning degeneration potentially resulting in misdiagnoses and staging inaccuracies [12,13]. In addition, it is not easy to perform an LB in clinical settings due to its invasiveness. To overcome these shortcomings, several noninvasive methods have been studied. Although various blood tests, such as the fatty liver index test, SteatoTest, and NAFLD score, and imaging studies are currently being examined [14–17], their accuracy has been insufficient. Recently, the controlled attenuation parameter (CAP) determined by the Fibroscan^®^ device (EchoSens, Paris, France) has been introduced as a simple method to assess hepatic steatosis [18–20]. According to previous studies, transient elastography (TE) has high accuracy and reproducibility when used to assess liver fibrosis [21,22]. In a recent study, CAP was also reported as an accurate factor with which to estimate hepatic steatosis [23].

Thus, this study aimed to investigate whether LS and CAP, assessed using TE, could discriminate patients with NASH from those with NAFLD and develop and validate a TE-based NASH prediction model.

## Patients and Methods

### Patients

Between November 2011 and December 2013, a total of 211 patients suspected of NAFLD who underwent LB with concomitant TE on the same day were recruited from five tertiary centers in South Korea.

Based on our exclusion criteria, patients with inappropriate LS values (failure of LS measurement or invalid LS value) were excluded. Additional exclusion criteria were as follows: (1) chronic hepatitis B or C; (2) chronic alcohol ingestion in excess of 40 g/day for more than 5 years; (3) the presence of autoantibodies; (4) missing clinical data; (5) small LB samples smaller than 15 mm in length, or (6) right-sided heart failure.

The database for our cohort included information on patient demographics, laboratory results, and LS and CAP values at the time of enrollment. A trained medical reviewer from each institute collected patient data from medical charts. The study was performed in accordance with the ethical guidelines of the 1975 Declaration of Helsinki. This study was approved by the Institutional Review Board from each institution (S1 Fig). Written informed consent was not required due to the retrospective nature of the study.

### Measurement of LS and CAP

LS and CAP measurements were performed on the same day as LB after fasting for at least 8 hours. LS measurements from TE were performed on the right lobe of the liver through the intercostal space of patients lying in the dorsal decubitus position with the right arm in maximal abduction [24]. TE was performed by one experienced technician blind to clinical patient data. The interquartile range (IQR) was defined as the index of the intrinsic variability of LS and CAP values corresponding to the interval of the LS and CAP results containing 50% of the valid measurements between the 25^th^ and 75^th^ percentiles. The median value of successful measurements was selected as representative of the LS and CAP values of subjects. The CAP measured ultrasonic attenuation at 3.5 MHz using signals acquired from TE [25]. As an indicator of variability, the ratios of the IQR of LS and CAP values to the median values (IQR/M and IQR/M_CAP_, respectively) were calculated. In this study, only procedures with at least 10 valid shots, a success rate of at least 60%, and an IQR/M of LS value less than 0.3 were considered reliable and used for statistical analysis.

### LB and diagnosis of NAFLD and NASH

All patients underwent ultrasound-guided percutaneous LB. The LB specimens were fixed in formalin and embedded in paraffin, and 4-μm thick sections were subjected to hematoxylin-eosin and Masson’s trichrome staining. All liver tissue samples were evaluated by an experienced hepatologist from each hospital who had no information about the clinical data of the study population. Liver specimens of 15 mm or longer in length or specimens in which the pathologist had confirmed their suitability for statistical analysis were regarded as reliable for assessing the grade and stage of hepatitis [26]. The definition of NAFLD requires (a) evidence of hepatic steatosis, either by imaging or histology, and (b) no cause for secondary hepatic fat accumulation [6]. NASH was defined as the presence of steatosis and inflammation with ballooning regardless of fibrosis [6]. Histological scoring was performed according to the NASH Clinical Research Network System [27]. Steatosis was assessed as the percentage of hepatocytes containing lipid droplets and categorized according to the NAFLD Activity Score (S0, <5%, S1, 5–33%, S2, 34–66%, and S3, >66%). Fibrosis was staged from 0 to 4: F0 = absence of fibrosis, F1 = perisinusoidal or portal, F2 = perisinusoidal and portal/periportal, F3 = septal or bridging fibrosis, and F4 = cirrhosis.

### Statistical anaylses

Data are expressed as the mean ± SD, median (range), or n (%), as appropriate. Variables were examined with the Student’s *t-*test (or Mann-Whitney test, if appropriate) and chi-square test (or Fisher’s exact test, if appropriate). Spearman’s correlation analysis was calculated to evaluate the correlations not only between LS and fibrosis but also between CAP and steatosis. In addition, box plots were used to show the LS and CAP distributions according to histologic fibrosis and steatosis grade. To evaluate the diagnostic accuracy of CAP for assessing hepatic steatosis, areas under the receiver operating characteristic curves (AUROCs) were calculated and compared using the method of Delong et al. [27]. Univariate and multivariate logistic regression analyses were performed for evaluating independent predictors of NASH. The optimal cut-off values were determined to maximize the sum of sensitivity and specificity, corresponding positive predictive values (PPVs), and negative predictive values (NPVs).

The CLA score was created using a set of clinical factors that had the best prognostic performance in the multivariable analysis. The adjusted odds ratio (OR) of each risk predictor was divided by the OR for LS values greater than 7 kPa and rounded to an integer value to generate each score. The risk-scoring model was the sum of each score assigned to each key variable. The bootstrap method, in which 1,000 random samples were drawn to replace the original dataset, was used to assess discriminatory power, and coefficients were recalculated in each bootstrap sample. All statistical analyses were assessed using the Statistical Package for Social Science (SPSS version 20.0, Armonk, NY, USA). A *P* value less than 0.05 was considered statistically significant.

## Results

### Baseline characteristics

Based on our exclusion criteria, three subjects with inappropriate LS values were excluded. Among patients with reliable LS values, an additional 25 were excluded. Finally, 183 subjects underwent statistical analysis.

The baseline characteristics of the study subjects are summarized in **Table 1**. The mean age was 40.6 years, and male gender was predominant (n = 111; 60.7%). Metabolic syndrome was identified in 40 (21.9%) patients. The prevalence of hypertension and diabetes mellitus was 87.4% (n = 160) and 14.2% (n = 26). Mean body mass index (BMI) and alanine aminotransferase (ALT) levels were 27.9 kg/m^2^ and 87.7 IU/L, respectively.

**Table 1:** Baseline characteristics of study population.

Variables	All (n = 183)	Non-NASH (n = 89)	NASH (n = 94)	*P* values	
Demographic variables					
Age (years)	40.6 ± 14.4	64.8 ± 81.0	80.0 ± 65.4	0.218	
Male gender	111 (60.7)	56 (62.9)	55 (58.5)	0.544	
Hypertension	160 (87.4)	5 (5.6)	76 (80.9)	0.006	
Diabetes mellitus	26 (14.2)	9 (10.1)	77 (81.9)	0.124	
Metabolic syndrome	40 (21.9)		78 (87.6)	84 (89.4)	0.002
Current smoking	32 (17.5)		9 (10.1)	8 (8.5)	<0.001
Body mass index (kg/m^2^)	27.9 ± 4.3		26.1 ± 3.8	29.0 ± 4.3	<0.001
Waist circumference (cm)	91.5 ± 16.2		88.4 ± 11.6	91.9 ± 16.8	0.602
Laboratory variables					
Total cholesterol (mg/mL)	195.7 ± 41.2		183.0 ± 35.1	204.0 ± 42.9	0.002
Triglycerides (mg/mL)	167.8 ± 102.0		155.1 ± 125.8	175.4 ± 84.4	0.246
HDL-cholesterol (mg/mL)	43.7 ± 11.5		44.6 ± 11.7	43.2 ± 11.5	0.59
LDL-cholesterol (mg/mL)	129.6 ± 31.0		105.8 ± 24.1	140.4 ± 27.7	<0.001
Aspartate aminotransferase (IU/L)	59.0 ± 58.9		37.3 ± 44.0	79.6 ± 64.0	<0.001
Alanine aminotransferase (IU/L)	87.7 ± 108.2		46.9 ± 57.0	126.4 ± 129.3	<0.001
Total bilirubin (mg/dL)	0.2 ± 0.4		0.2 ± 0.5	0.2 ± 0.4	0.877
Gamma glutamyltransterase (IU/L)	74.4 ± 71.7		64.8 ± 81.0	80.0 ± 65.4	0.218
Serum albumin (g/dL)	4.4 ± 0.5		4.4 ± 0.4	4.4 ± 0.5	0.336
Platelet count (10^3^/uL)	231 ± 61		225.8 ± 60.0	234.5 ± 61.3	0.386
Prothrombin time (INR)	1.0 ± 0.1		1.0 ± 0.1	1.0 ± 0.1	0.822
Alpha fetoprotein (ng/mL)	5.2 ± 3.6		3.8 ± 1.9	5.6 ± 3.9	0.039
Fasting glucose (mg/mL)	108.7 ± 34.0		109.0 ± 27.9	108.5 ± 37.4	0.933
Liver Histology					
NAS score 0-2/3-4/ ≥5	67(36.6)/22(12.0)/94(51.4)		67(75.3)/11(12.4)/11(12.4)	0(0)/11(11.7)/83(88.3)	<0.001
Steatosis 0/1/2/3	9(4.9)/76(41.5)/65(35.5)/33(18.0)		9(10.1)/55(61.8)/17(19.1)/8(9.0)	0(0)/21(22.3)/48(51.1)/25(26.6)	<0.001
Lobular inflammation 0/1/2/3	76(41.5)/61(33.3)/42(23.0)/4(2.2)		76(85.4)/11(12.4)/1(1.1)/1(1.1)	0(0)/50(53.2)/41(43.6)/3(3.2)	<0.001
Hepatocyte ballooning 0/1/2	85(46.4)/72(39.3)/26(14.2)		85(95.5)/2(2.2)/2(2.2)	0(0)/70(74.5)/24(25.5)	<0.001
Fibrosis 0/1/2/3/4	76(41.5)/61(33.3)/18(9.8)/11(6.0)/17(9.3)		74(83.1)/5(5.6)/1(1.1)/1(1.1)/8(9.0)	2(2.1)/56(59.6)/17(18.1)/10(10.6)/9(9.6)	<0.001
Liver stiffness measurement					
Liver stiffness value (kPa)	8.6 ± 6.3		7.3 ± 6.3	9.8 ± 6.0	0.006
Interquartile range (kPa)	1.1 ± 1.0		1.0 ± 1.0	1.2 ± 1.3	0.371
Interquartile range/ median (%)	7.6 ± 6.2		7.6 ± 6.0	7.6 ± 6.1	0.518
Controlled attenuation parameter					
CAP value (dB/m)	282.7 ± 52.6		259.4 ± 54.2	304.8 ± 40.3	<0.001
Interquartile range (dB/m)	26.7 ± 10.1		27.1 ± 11.0	26.3 ± 9.1	0.783
Interquartile range/ median (%)	9.4 ± 4.4		9.7 ± 5.1	9.1 ± 3.5	0.635

Variables are expressed as mean ± SD (range) or n (%).

HDL, high-density lipoproteins; LDL, low-density lipoproteins; NASH, non-alcoholic steatohepatitis; NAS score; non-alcoholic fatty liver disease activity score; kPa, kilopascal; CAP, controlled attenuation parameter.

When compared between NASH (n = 94, 51.4%) and non-NASH (n = 89, 48.6%) group, ALT level, LS value, and CAP value were significantly higher in patients with NASH than those with non-NASH (all *P*<0.05). Patients with NASH showed higher proportion of hypertension and metabolic syndrome, lower proportion of current-smokers, higher BMI, higher LDL-cholesterol, and higher AST than those of patients with non-NASH (all *P*<0.05).

### Liver histology

Distributions for steatosis, lobular inflammation, hepatocyte ballooning, and fibrosis in the study population are shown in **Table 1**. Among all subjects, 89 (48.6%) patients had non-NASH, and 94 (51.4%) had NASH. Patients with NAFLD Activity Score (NAS) score 0–2, 3–4, and greater than 5 were 67 (36.6%), 22 (12.0%), and 94 (51.4%), respectively.

### CAP and LS values according to steatosis and fibrosis stages

**Fig 1:**
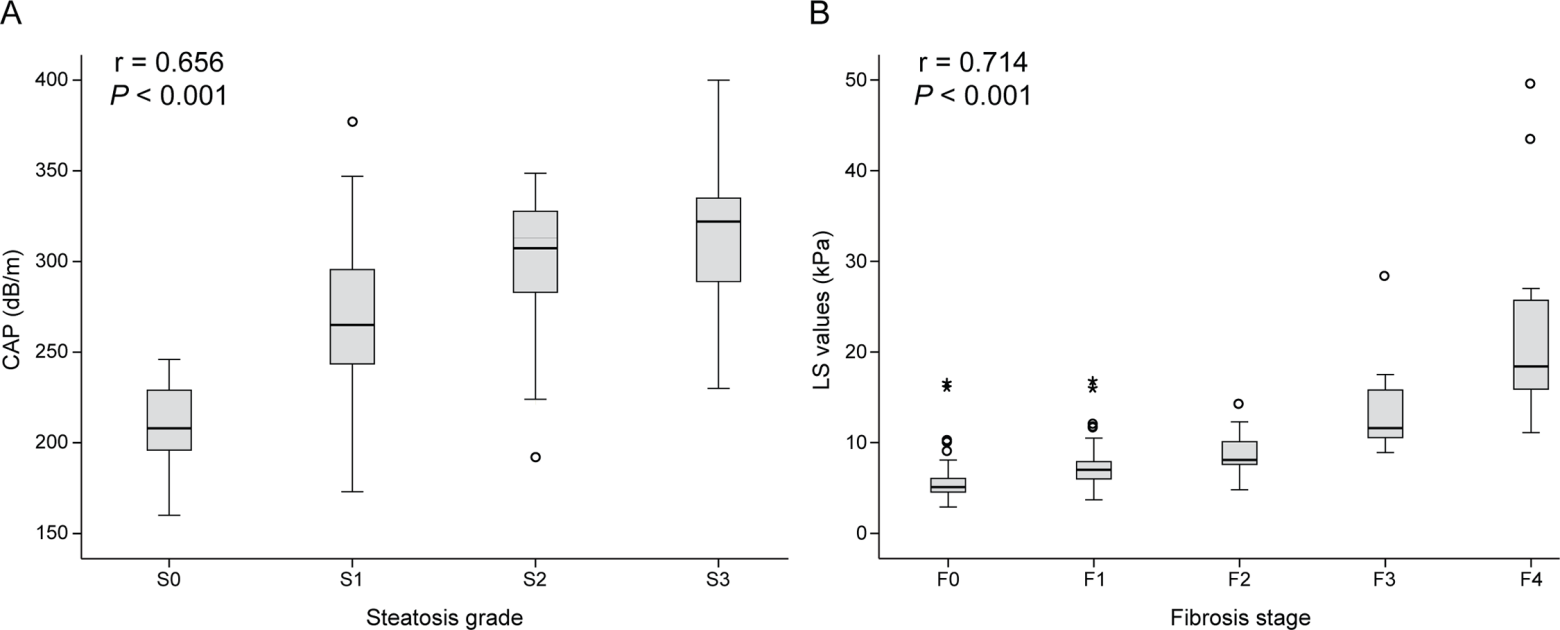
The distribution of LS and CAP values according to histologic fibrosis and steatosis grade. The bar lines mean the range of each grade of steatosis and fibrosis. LS, liver stiffness; CAP, controlled attenuation parameter.

The mean CAP and LS values were 282.7 dB/m [median 285.0 dB/m (range, 160.0–400.0)] and 8.6 kPa [median 6.5 kPa (range, 2.9–49.6)], respectively. The distribution of CAP and LS values are described in **Fig 1**. There was a significant correlation between CAP and histologic steatosis (*r* = 0.656, *P*<0.001). The median CAP value was 208.0 (range, 160.0–246.0) for S0, 265.0 (range, 173.0–377.0) for S1, 313.0 (range, 192.0–350.0) for S2, and 322.0 (range, 230.0–400.0) for S3. In addition, LS values increased with histological fibrosis stage (*r* = 0.714, *P*<0.001). The median LS value was 5.1 (range, 2.9–16.6) for F0, 7.0 (range, 3.7–16.8) for F1, 8.1 (range, 4.8–14.3) for F2, 11.6 (range, 8.9–28.4) for F3, and 18.4 (range, 11.1–49.6) for F4.

### Determination of optimal cut-off CAP value, LS values, and ALT level

The optimal cut-off CAP values and their diagnostic indices are shown in **Table 2**. The cut-off CAP values were 247 dB/m for S0 vs. S1–3 (AUROC 0.953, 95% CI 0.925–0.981), 280 dB/m for S0–1 vs. S2–3 (AUROC 0.855, 95% CI 0.799–0.912), and 300 dB/m for S0–2 vs. S3 (AUROC 0.726, 95% CI 0.645–0.807). In addition, the optimal cut-off LS values and their diagnostic indices are shown in Table 3. The cut-off LS values were 6.7 kPa for F0 vs. F1–4 (AUROC 0.855, 95% CI 0.798–0.911), 8.0 kPa for F0–1 vs. F2–4 (AUROC 0.892, 95% CI 0.835–0.950), 9.0 kPa for F0–2 vs. F3–4 (AUROC 0.970, 95% CI 0.949–0.992), and 11.0 kPa for F0–3 vs. F4 (AUROC 0.974, 95% CI 0.953–0.995). The optimal cut-off value for ALT to predict the presence of steatosis was 57.5 IU/L (sensitivity 75.5%, specificity 77.5%). For easy interpretation and further statistical analysis, we adopted 250 dB/m, 7.0 kPa, and 60.0 IU/L as our cutoff values, respectively.

**Table 2:** Diagnostic performance of CAP for assessing hepatic steatosis in patients with NAFLD.

Variable	S0 (n = 9) vs. S1-3 (n = 174)	S0-1 (n = 85) vs. S2-3 (n = 98)	S0-2 (n = 150) vs. S3 (n = 33)
AUROC (95% CI)	0.953 (0.925–0.981)	0.855 (0.799–0.912)	0.726 (0.645–0.807)
Optimal CAP cutoff value (dB/m)	247	280	300
Sensitivity, % (95% CI)	88.2 (83.1–93.3)	84.7 (77.6–91.8)	72.7 (57.5–87.9)
Specificity, % (95% CI)	100.0	80.0 (71.4–88.6)	60.7 (51.1–70.2)
Positive predictive value, % (95% CI)	100.0	83.0 (75.6–90.4)	28.9 (19.1–38.7)
Negative predictive value, % (95% CI)	62.5 (48.8–76.2)	81.9 (73.6–90.2)	91.0 (85.4–96.6)

CAP, controlled attenuation parameter; NAFLD, non-alcoholic fatty liver disease; AUROC, area under the ROC curve; CI, confidence interval.

**Table 3:** Diagnostic performance of LS for assessing liver fibrosis in patients with NAFLD

Variable	F0 (n = 76) vs. F1-4 (n = 107)	F0-1 (n = 137) vs. F2-4 (n = 46)	F0-2 (n = 155) vs. F3-4 (n = 28)	F0-3 (n = 166) vs. F4 (n = 17)
AUROC (95% CI)	0.855 (0.798–0.911)	0.892 (0.835–0.950)	0.970 (0.949–0.992)	0.974 (0.953–0.995)
Optimal LS cutoff value (kPa)	6.7	8.0	9.0	11.0
Sensitivity, % (95% CI)	66.4 (57.5–75.2)	82.6 (71.7–93.6)	96.4 (89.6–1.03)	100.0 (100.0–100.0)
Specificity, % (95% CI)	84.9 (77.9–92.0)	84.7 (78.3–91.0)	85.8 (79.9–91.7)	89.8 (84.9–94.6)
Positive predictive value, % (95% CI)	88.0 (80.9–95.0)	64.4 (52.1–76.6)	55.1 (41.2–69.0)	44.7 (28.9–60.5)
Negative predictive value, % (95% CI)	62.6 (53.1–72.2)	93.5 (89.2–98.9)	99.3 (97.8–100.7)	100.0 (100.0–100.0)

LS, liver stiffness; NAFLD, non-alcoholic fatty liver disease; AUROC, area under the ROC curve; CI, confidence interval

### Independent predictors of NASH and development of CLA score

As shown in **Table 4**, univariate analysis identified that CAP values greater than 250 dB/m, LS values greater than 7 kPa, ALT level greater than 60 IU/L, hypertension, current smoker, and presence of metabolic syndrome were significant for predicting NASH (all *P*<0.05). On subsequent multivariate analysis, a *C*AP value greater than 250 dB/m (OR 4.133, 95% CI 1.037–16.470), an *L*S value greater than 7 kPa (OR 3.399, 95% CI 1.295–8.291), and an *A*LT level greater than 60 IU/L (OR 7.557, 95% CI 2.997–19.059) independently predicted NASH. In addition, a *C*AP value greater than 250 dB/m (AUROC 0.743, 95% CI 0.669–0.816, sensitivity 95.7%, specificity 49.4%), an *L*S value greater than 7 kPa (AUROC 0.751, 95% CI 0.677–0.824, sensitivity 86.2%, specificity 58.4%), and an *A*LT level greater than 60 IU/L (AUROC 0.829, 95% CI 0.689–0.832, sensitivity 73.4%, specificity 78.7%) showed the best AUROC. Based on this result, we developed a new risk-scoring model for discriminating NASH. The adjusted OR of each risk predictor was divided by the OR for LS value greater than 7kPa and was rounded to an integer value to generate each score. Then, the CLA score was calculated by the sum of each score assigned to each key variable (**Table 4**). The prevalence of NASH significantly increased with higher CLA score and CLA risk stratification (**Table 5**).

**Table 4:** Independent predictors of NASH and corresponding rounded risk score for CLA score.

Variable	Univariate	Multivariate			CLA scoring
	*P* value	*P* value	Odds ratio	95% CI	
CAP value >250 dB/m	<0.001	0.044	4.133	1.037–16.470	1
LS value >7 kPa	<0.001	0.013	3.399	1.295–8.291	1
ALT >60 IU/L	<0.001	<0.001	7.557	2.997–19.059	2
Age	0.163				
Hypertension	0.009	0.414	2.003	0.378–10.610	
Diabetes mellitus	0.811				
Current smoking	<0.001	0.090	0.618	0.353–1.079	−
Metabolic syndrome	0.003	0.555	1.539	0.368–6.431	−
Body mass index (kg/m^2^)	0.054				
LDL-cholesterol (mg/mL)	0.060				
Aspartate aminotransferase (IU/L)	0.283				
Alpha fetoprotein (ng/mL)	0.146				

NASH, nonalcoholic steatohepatitis; CI, confidence interval; CAP, controlled attenuation parameter; LS, liver stiffness; ALT, alanine aminotransferase.

**Table 5:** CLA score and corresponding NASH prevalence.

Risk stratification	CLA score	Scoring			NASH (n)	Total (n)	NASH in each score (%)	NASH in each risk group (%)
		By CAP values (>250 dB/m)	By LS value (>7 kPa)	By ALT level(>60 IU/L)				
Low risk	0	0	0	0	2	40	5.0	5.0
Intermediate	1	0	1	0	2	6	29.0	42.1
risk		1	0	0	7	25		
	2	1	1	0	14	24	57.7	
		0	0	2	1	2		
High risk	3	1	0	2	23	32	70.6	79.1
		0	1	2	1	2		
	4	1	1	2	44	52	84.6	

NASH, nonalcoholic steatohepatitis; CAP, controlled attenuation parameter; LS, liver stiffness; ALT, alanine aminotransferase

### Diagnostic performance of CLA score

**Fig 2:**
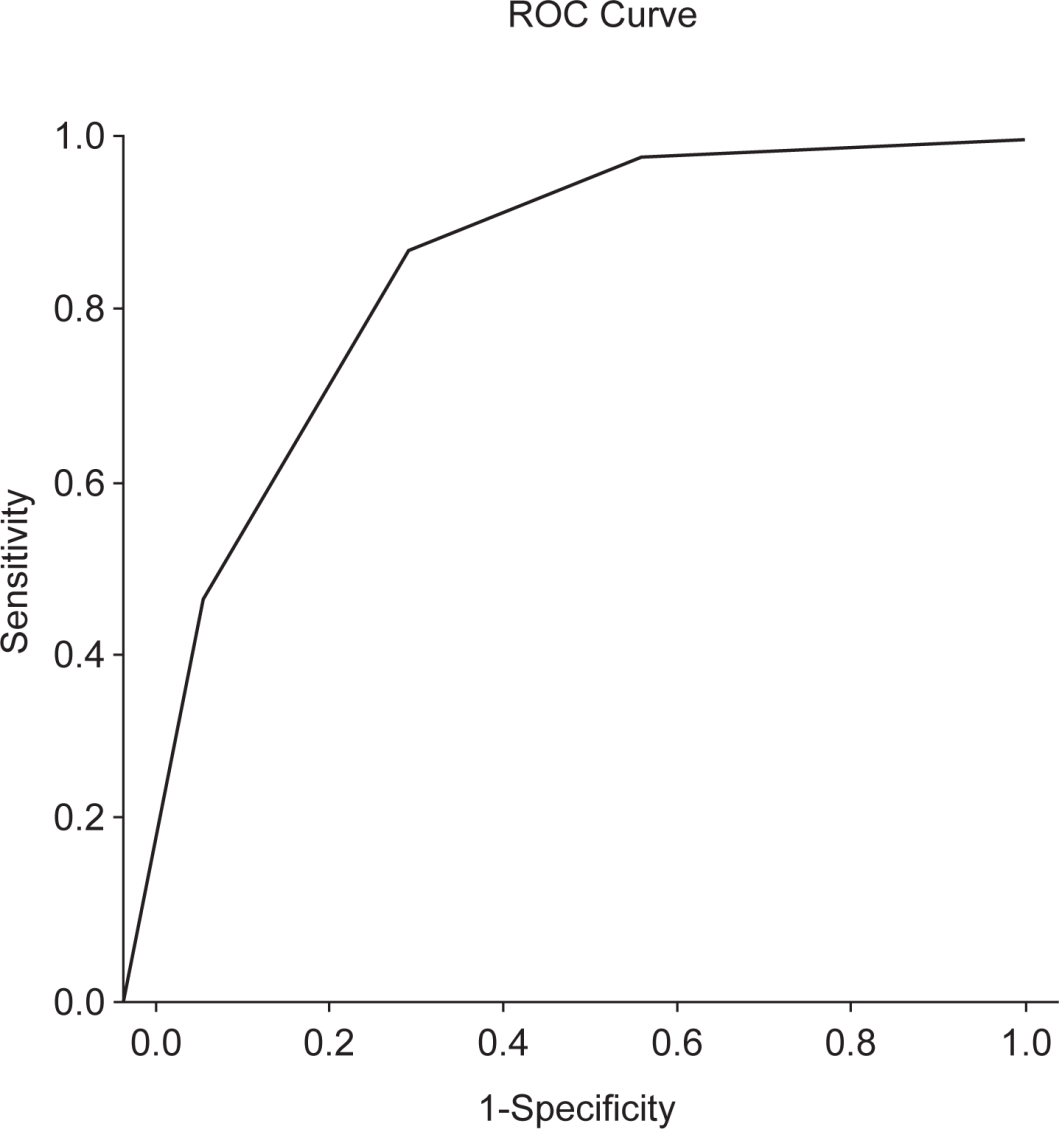
Internal validation of CLA scores for predicting NASH. AUROC, area under receiver operating characteristic curve; CI, confidence interval.

The AUROC of the CLA score to predict NASH was 0.812 (95% CI 0.724–0.880), which was significantly higher than that of NAFLD fibrosis score (NFS), which has been widely used as a scoring system to separate NAFLD patients with and without advanced fibrosis (AUROC = 0.618; 95% CI 0.472–0.796) [28]. To assess discriminatory power, we used the bootstrap method. As shown in **Fig 2**, the AUROCs remained largely unchanged between iterations, with an average AUROC of 0.833 (95% CI 0.740–0.893).

## Discussion

In this study, we found that the accuracy of TE to predict the degree of liver fibrosis (AUROC 0.855–0.974) and steatosis (AUROC 0.726–0.953) was acceptable, consistent with previous studies [29–31], establishing a new NASH prediction model, named “CLA score,” using three independent predictors (CAP score, LS value, and ALT level) that were identified from multivariate analysis. The accuracy of the CLA score was acceptable (AUROC = 0.812), and its accuracy remained largely unchanged between iterations, with an average AUROC of 0.833 in internal validation. Using this CLA score, patients with NAFLD could be stratified into three groups with significantly different risk of NASH (prevalence from 5.0% in the low-risk group to 79.1% in the high-risk group).

Our study has several strengths. First, the CLA score is derived from easy-to-access laboratory tests and TE results. Although TE is still limited to tertiary academic hospitals, the use of TE has become more popular due to the extensive validation of its clinical usefulness and proposed guidelines [32]. If TE becomes widely available, the CLA model might help physicians assess the risk of disease progression to NASH among asymptomatic patients with NAFLD who are vulnerable to the silent progression of advanced liver disease. Second, for more accurate prediction using the CLA model, we used CAP as a constituent variable to assess the degree of hepatic steatosis, using a highly reproducible and operator-independent technique unlike other image modalities such as ultrasound [33]. In addition, because CAP is calculated based on the same volume of the liver (100-times larger than LB) as LS, CAP might be less influenced by sampling error [34]. Third, TE showed higher diagnostic performance among other noninvasive assessment tools for liver fibrosis, including several fibrosis scoring systems of patients with NAFLD [35,36]. Thus, the CLA model is the first scoring system that can assess the degree of liver fibrosis and steatosis simultaneously using TE, which are the most important sequelae in the pathogenesis of NAFLD.

Unlike a previous study by Lupşor-Platon et al. (AUROCs of 0.813 for ≥S1, 0.822 for ≥S2, and 0.838 for S3) [30], AUROCs in our study tended to decrease as steatosis grade increased (AUROCs of 0.953 for ≥S1, 0.855 for ≥S2, and 0.726 for S3). Although the exact reason for this phenomenon is unclear, the skewed distribution of patients with each hepatic steatosis grade (only 33 patients with S3, 18%) might have influenced our results. However, the accuracy of CAP is similar to that of another study by Chon et al. [29] (AUROCs of 0.885 for ≥S1, 0.894 for ≥S2, and 0.800 for S3), which also showed a decrease in CAP AUROCs as hepatic steatosis grade increased. On the other hand, the cut-off CAP value in our study for each steatosis grade was 247 dB/m for ≥S1, 280 dB/m for ≥S2, and 300 dB/m for S3. Although previous studies proposed quite similar cut-off values to those in our study (260 dB/m for ≥S1, 285 dB/m for ≥S2, and 294 dB/m for S3) [29,30], the cut-off CAP values differ by ethnicity and the distribution of each steatosis grade [22,29,37]. In our study, the cut-off LS values for each fibrosis stage were 8.0 kPa for ≥F2, 9.0 kPa for at ≥F3, and 11.0 kPa for F4, which are similar to those from other studies that recruited patients with NAFLD [31,36,38].

Predictors of advanced fibrosis among NASH patients vary by study, but commonly include age, metabolic syndrome-associated factors (obesity, the presence of insulin resistance, diabetes mellitus, hypertension, and hypertriglycemia), and elevated ALT level [7,39,40]. However, only three factors (CAP value >250 dB/m, LS value >7 kPa, and ALT level >60 IU/L) were independent predictors of NASH in our study. When we used NASH as the end-point of three variables, the cut-off value of CAP (245 dB/m, AUROC 0.743) and ALT level (59.5 IU/L, AUROC 0.829) were not largely changed. However, the cut-off value of LS was slightly lowered (5.8 kPa; AUROC 0.751). However, when we used LS value >6 kPa, not >7 kPa, as the cut-off value to establish another CLA model, AUROC values of original CLA model using LS value >7 kPa (AUROC 0.812) and new CLA model using LS value >6 kPa (AUROC 0.810) was still similar. Metabolic syndrome, diabetes mellitus, and hypertension have been already reported as important factors for NASH development [39,40], all of which showed borderline statistical significance in our study (*P*<0.1). However, the influence of metabolic syndrome, diabetes mellitus, and hypertension was attenuated in multivariate analysis, perhaps due to the overwhelming influence of the strong association between the risk of NASH and the three constituent variables of the CLA score. Indeed, in other studies that did not consider the influence of hepatic fibrosis and steatosis, metabolic syndrome, diabetes mellitus, and hypertension were selected as independent predictors of NASH [41,42]. Further studies with larger sample size might be required to investigate whether the addition of metabolic syndrome, diabetes mellitus, and hypertension can enhance the overall prediction accuracy of the CLA score.

To date, several prediction models to distinguish subjects with and without advanced fibrosis in NAFLD are available [28,43,44]. Of these, NFS is the most commonly used, composed of six variables including age, BMI, diabetes, AST/ALT ratio, platelet count, and serum albumin [28]. Using NFS, it was reported that LB can be avoided in 75% of patients (28). However, when NFS applied to our cohort, AUROC was only 0.618 (PPV 75.0% and NPV 60.3%), perhaps due to the different proportions of advanced steatosis and BMI compared to Western studies [35,45]. Although issues related to the cost of TE to obtain CLA score still remain, CLA score assessed using TE (AUROC 0.812, PPV 84.0%, and NPV 72.5%) might be superior to NFS in Asian patients with NAFLD.

For simple clinical applications, we stratified our study population into three different risk groups according to CLA score (low, intermediate, and high-risk groups). NASH was present in 79.1% of patients at high risk (CLA score ≥3.0), whereas only 5.0% patients were at low risk (CLA score <1.0). Therefore, a high CLA score implies that a patient has a higher risk of NASH and requires more intensive care, whereas patients in low-risk groups may merely need careful follow up and monitoring of CLA score for early identification of disease progression.

There are a few limitations of our study. First, although this study was designed as a multicenter study, the overall sample size of our study was still relatively small. Further studies including larger cohorts are required for external validation. Second, study participants from major Korean tertiary hospitals may not accurately represent the NAFLD situation in Korea. Patients in our study might suffer from more advanced disease than the general population in primary care settings, resulting in selection bias. Third, 20.9% (18/86) of patients with CLA score-defined high risk did not have NASH, but only NAFL. Considering this unsatisfactory accuracy, further studies refining our CLA model should be followed. Finally, because pathological interpretations at each institute were permitted, our study might not be free from errors in the assessments of the degrees of liver fibrosis and steatosis.

In conclusion, CAP and LS can be used as reliable, noninvasive markers for grading steatosis and fibrosis in Korean patients with NAFLD. A TE-based, simple-to-use scoring model (CLA score) was created and subsequently validated, showing acceptable accuracy in discriminating NASH patients from those with simple steatosis. Using the CLA score, clinicians can diagnose or exclude NASH noninvasively and decide to perform LB in patients requiring a histological diagnosis among patients with NAFLD.

## Supporting Information

S1 Fig This is the report of Institutional Review Board approval. (PDF)

## Abbreviations

NAFLD: nonalcoholic fatty liver disease
NAFL: nonalcoholic fatty liver
NASH: nonalcoholic steatohepatitis
LB: liver biopsy
CAP: controlled attenuation parameter
TE: transient elastography
IQR: interquartile range
AUROC: area under the receiver operating characteristic curve
PPV: positive predictive value
NPV: negative predictive value
OR: odd ratio
BMI: body mass index
ALT: alanine transaminase
NAS: nonalcoholic fatty liver disease activity score
