# Supplementary material for: Discrimination of Nonalcoholic Steatohepatitis Using Transient Elastography in Patients with Nonalcoholic Fatty Liver Disease
Source: PLoS One. 2016 Jun 10;11(6):e0157358. doi: 10.1371/journal.pone.0157358 (PMC4902201; doi:10.1371/journal.pone.0157358)
Supplement: S1 Fig — (PDF) [file pone.0157358.s001.pdf]

Report of Institutional Review Board

Requester **: Hye-won Lee**

This letter is to inform you of the results of your confidential.

Type of Review	■ first approval □ approval with condition □ complement □ return<br>□ objection □ change on protocol □ interim report □ end of study report<br>□ final report □ cancellation of protocol □ others			
IRB No.	**4-2015-0520**		Date of approval	July 28th, 2015
Title of Proposal	The usefulness of liver stiffness and controlled attenuation parameter measured by transient elastography in patients with chronic liver disease			
	Protocol No.	–	Version No.	-
Investigator	Principal Investigator: Dr. Beom-kyung Kim / Assistant Clinical Professor / Institute of Gastroenterology and Hepatology / Division of Gastroenterology, Department of Internal Medicine / Yonsei University College of Medicine			
Generic name	-		Brand Name	-
Phase	□ Phase I □ Phase II □ Phase III □ Phase IV<br>□ Biological equivalence test ■ Others			
Proposed period of study	Approval date of IRB ~ for 60 months			
Contents of Review	※ Lists of initial review (reviewed by E-IRB system)<br>1. Application Form<br>2. Retrospective Study Protocol<br>3. Case Report Form<br>4. Informed Consent Waiver Form<br>5. CV for Investigator			
Date of Review	July 28th, 2015			
Result of Review	■ Approved (Exemption) □ Approval with condition □ Require to re-reviewed<br>□ Disapproval			
Comment	none			

Severance Hospital IRB is organized and operates according to ICH-GCP and the applicable laws and regulations

July 28th, 2015

Seung Min Kim, MD, PhD
Chairperson of Institutional Review Board
Severance Hospital
Yonsei University, College of Medicine
Seoul 120-752, Korea
